# Polymer Electrolyte Membranes Based on Nafion and a Superacidic Inorganic Additive for Fuel Cell Applications

**DOI:** 10.3390/polym11050914

**Published:** 2019-05-22

**Authors:** Lucia Mazzapioda, Stefania Panero, Maria Assunta Navarra

**Affiliations:** Department of Chemistry, Sapienza University of Rome. Piazzale Aldo Moro 5, 00185 Rome, Italy; lucia.mazzapioda@uniroma1.it (L.M.); stefania.panero@uniroma1.it (S.P.)

**Keywords:** mesoporous sulfated titania, nanocomposite polymer electrolytes, PEM fuel cells

## Abstract

Nafion composite membranes, containing different amounts of mesoporous sulfated titanium oxide (TiO_2_-SO_4_) were prepared by solvent-casting and tested in proton exchange membrane fuel cells (PEMFCs), operating at very low humidification levels. The TiO_2_-SO_4_ additive was originally synthesized by a sol-gel method and characterized through x-ray diffraction (XRD), scanning electron microscopy (SEM), thermogravimetric analysis (TGA) and ion exchange capacity (IEC). Peculiar properties of the composite membranes, such as the thermal transitions and ion exchange capacity, were investigated and here discussed. When used as an electrolyte in the fuel cell, the composite membrane guaranteed an improvement with respect to bare Nafion systems at 30% relative humidity and 110 °C, exhibiting higher power and current densities.

## 1. Introduction

Fuel cells are electrochemical devices with high-energy conversion efficiency, minimized pollutant emission and other advanced features. Among the different types of fuel cells, including alkaline fuel cell (AFC), phosphoric acid fuel cell (PAFC), direct-methanol fuel cell (DMFC), molten carbonate fuel cell (MCFC) and solid oxide fuel cell (SOFC), the proton exchange membrane fuel cell (PEMFC) is attractive both for automobile and stationary applications [1,2]. The conventional design of PEMFCs consists of anodic and cathodic compartments, separated by a proton exchange membrane. The most used as a proton exchange membrane for these devices is Nafion (Du Pont), a perfluorosulfonic acid polymer [3], thanks to its high proton conductivity, suitable mechanical properties, chemical and electrochemical stability, low fuel permeability and electronic insulation [4,5]. The major issue for this membrane is the decrease of its conductivity under desirable operating conditions, i.e., low relative humidity (RH) and temperature higher than 80 °C [6,7]. Indeed, on one hand, the electrode kinetics will be enhanced and the overpotential will be reduced when the temperature is increased. On the other side, at low RH the water management will be simpler with respect to devices working fully humidified [8]. 

According to several studies [9,10], a strategy to overcome these limits is to modify the polymer matrix with inorganic additives, that are able to improve the low RH and high temperature performances of PEMFCs [11,12]. These additives may be metal oxide nanoparticles, such as SiO_2_, TiO_2_, ZrO_2_ or functionalized inorganic materials, such as sulfated metal oxides [13,14]. The presence of acidic hygroscopic additives ensures better hydration and proton conductivity of the membranes, allowing working at the targeted conditions described above [15,16]. These composite membranes also decrease the cross-over of the gases during fuel cell operations [17]. 

The family of sulfated metal oxide (S-MO_2_) is very attractive, thanks to the intrinsic super-acidity properties. It has been widely investigated by us to form nano-composite membranes with good performances at high temperature and low RH [18,19,20]. In the present work, we propose the use of mesoporous sulfated titanium oxide (TiO_2_-SO_4_), newly synthesized, as an additive in Nafion. With respect to other studies already reported by us, the additive here was prepared by a template-driven procedure, to control the structure of the particles. Taking advantage of the properties of TiO_2_-SO_4_, we demonstrate the applicability of the proposed composite membranes in PEMFCs at high temperature and low relative humidity.

## 2. Materials and Methods 

A sol-gel one-step synthesis was developed to obtain mesoporous TiO_2_-SO_4_, with hydrolysis and sulfation happening in a single step [21], then followed by a hydrothermal treatment. Titanium isopropoxide (Sigma Aldrich, St. Louis, MO, USA) was used as a precursor and Pluronic 123 copolymer (Sigma Aldrich, M_w_~5800) was used as a structure-directing agent in order to obtain a mesoporous compound. The TiO_2_-SO_4_ was prepared according to the following procedure: Pluronic 123 was added to sulfuric acid (H_2_SO_4_, 95%–97%, Sigma Aldrich, St. Louis, MO, USA) in vigorous stirring for 15 min at 35 °C. Subsequently, titanium isopropoxide was added to the mixture and after 15 min the solution was transferred in an autoclave and it was treated before at 30 °C for 20 h and then at 100 °C for 48 h. The solid product was filtered and calcinated for 3 h at 550 °C (heat rate 3 °C/min).

Nafion membranes were prepared by a solvent casting procedure, starting from Nafion wt. 5 % solution (E.W. 1100, Ion Power Inc, München Germany), where solvents (water and alcohols) were gradually replaced with N,N-dimethylacetamide (> 99.5%, Sigma Aldrich, St. Louis, MO, USA) at 80 °C [9]. For the composite membranes, the TiO_2_-SO_4_ was added to the final Nafion solution. A filler concentration of 2 wt %, 5 wt % and 7 wt %, with respect to the dry Nafion content, was chosen. The mixture obtained was casted on a Petri dish and dried at 80 °C. After the heating treatment, dry membranes were extracted and hot-pressed at 50 atm, 175 °C for 15 min. This method is needed to improve the thermal stability of the membranes and allows a preferable cohesion between ionic clusters [22]. The membranes were finally activated in boiling 3% w/w hydrogen peroxide (H_2_O_2_, 34.5%-36.5%, Sigma Aldrich, St. Louis, MO, USA), H_2_SO_4_ (0.5 M) and distilled water. 

Nano-composite membranes have been compared to plain Nafion systems prepared with the same procedure. All samples were stored in bi-distilled water.

X-ray diffraction analysis (XRD) was carried out to study the phases of the prepared TiO_2_-SO_4_ additive. The X-ray analysis was performed using a Rigaku D-Max Ultima + diffractometer, provided with a graphite monochromator, in the 2θ range 20–90°. The radiation used was the Kα of Cu. The crystallite size was obtained using Maud code. 

Nitrogen-adsorption experiments were used for the determination of pores size distributions and for the evaluation of the specific surface area of the inorganic powder by the Brunauer-Emmett-Teller (BET) equation, using a Micromeritics ASAP 2010. Before of the measurement, each sample needed a pre-treatment at 200 °C for 2 h in order to remove physisorbed water. Through the scanning electron microscopy (SEM, Phenom, Eindhoven, The Netherlands) analysis, the morphology and size of the inorganic additive were evaluated.

Thermal analysis were conducted by means of differential scanning calorimetry (DSC), using DSC821 instrument (Mettler-Toledo, Zaventem, Belgium), and by thermal gravimetric (TG) analysis, performed with a TGA/SDTA851 (Mettler-Toledo, Zaventem, Belgium). DSC was carried out on the membrane samples under nitrogen (N_2_) flux (60 mL/min) at a scan rate of 20 °C/min. Before DSC measurements, membrane samples were equilibrated at 100% relative humidity (RH) for two weeks. TG analysis was carried out on TiO_2_-SO_4_ powder sample under air flux (60 mL/min) at a scan rate of 5 °C/min.

The ion-exchange capacity (IEC), which expresses the amount of exchangeable protons, was evaluated by a titration method both for the powder and for all the membranes. Dry samples were immersed in sodium chloride (NaCl) aqueous solution and the exchanged protons were neutralized with a standard solution of sodium hydroxide (NaOH) 0.1 M [23].

For all the membranes the total water uptake (WU) was evaluated at room temperature by a gravimetric method, according to the following equation:

(1) $$W.U.=\frac{W_{\mathrm{wet}}-W_{\mathrm{dry}}}{W_{\mathrm{dry}}}\times 100$$

where W_wet_ is the weight of fully hydrated membranes, obtained by equilibrating the samples in a close container under saturated water atmosphere (100% RH) for two weeks. W_dry_ is the weight of dry membranes, measured after one night at 80 °C under vacuum. For both W.U. and IEC values, errors were evaluated with standard deviation of three different measurements.

Hydration number, known as λ, is a parameter that allows to define the number of water molecules for each acid group [24]. It was calculated by the following equation:

(2) $$\lambda =\;\frac{{\mathrm{mol}\;H}_{2}O}{\mathrm{mol}\;\mathrm{acid}\;\mathrm{groups}}=\;\frac{W.U.}{\mathrm{IEC}}\times \frac{10}{18}$$

Fuel cell tests were performed by using a compact system (850C, Scribner Associates Inc, Southern Pines, NC, USA) connected to a 5 cm^2^ cell fixture. The membrane electrode assembly (MEA) was prepared as follows: The surface of the electrodes (Basf, 0.5 mg Pt cm^−2^) was brushed with Nafion solution (5% w/w E.W. 1100, Ion Power Inc, München, Germany), resulting in ca. 0.4 mg dry Nafion cm^−2^ after the evaporation of the solvent. The membrane was hot-pressed between two electrodes at 120 °C and 10 atm for 7 min. The cell was fed by hydrogen (H_2_) and air with a backpressure of 1 atm. The humidification of the cell was accomplished by bubbling the fed gases through stainless steel cylinders incorporated in the compact system and containing distilled water. The temperature of the humidifiers, as well as that of the cell, was properly set to achieve the desired relative humidity. In situ electrochemical impedance spectroscopy (EIS) was performed with the 880 Impedance Analyzer in the 10 KHz–1 Hz frequency range. The amplitude of the sine wave was chosen to be 5% of the DC current present at 0.6 V cell voltage.

## 3. Results and Discussion

### 3.1. Characterization of Powder

**Figure 1:**
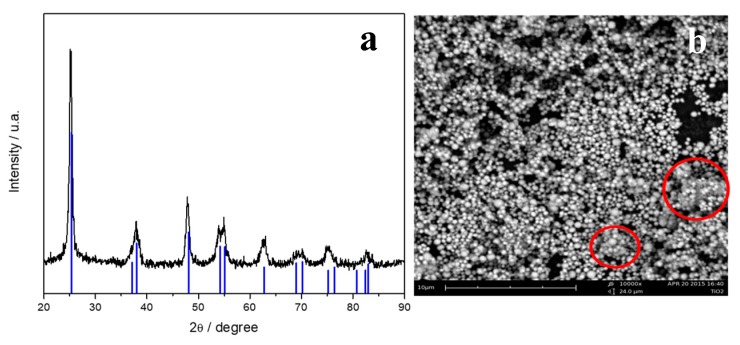
X-ray diffraction pattern (**a**) and SEM image (**b**) of TiO_2_-SO_4_ powder.

The XRD pattern of the titania powder is reported in Figure 1a. The sample resulted in a pure anatase phase, characterized by tetragonal I41/amd space group. Blue tick marks correspond to the anatase TiO_2_ (CIF number 96-900-8214). The mean crystallite size, determinate by the Maud code, is 18 ± 0.36 nm.

An SEM image of the TiO_2_-SO_4_ powder is reported in Figure 1b. It is evident that the method adopted leads to the development of spherical particles of the metal oxide. These particles have a homogeneous size distribution at the sub-micrometric range, with some possible aggregates, as highlighted by the red circles.

**Figure 2:**
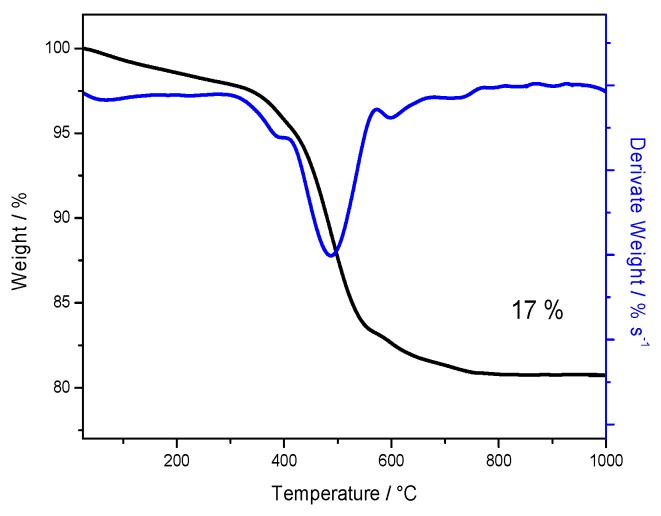
Thermo-gravimetric (black curve) and derivative thermo-gravimetric (DTG, blue curve) analysis of TiO_2_-SO_4_ powder.

In order to evaluate the amount of sulfate groups on the powder sample, a thermo-gravimetric analysis was carried out under air in a temperature range between 25 °C and 1000 °C and reported in Figure 2, both as thermo-gravimetric (TG) and derivative thermo-gravimetric (DTG) curves. Below 400 °C, dehydration and removal of chemisorbed hydroxyl groups occur. Another important mass loss is observed between 400 °C and 800 °C, due to the removal of sulfate groups, bonded on the surface of titanium oxide. This loss is ca. 17%, as reported also in Figure 2, revealing an important sulfation degree, much higher than others previously reported [9,12,19,21] and suggesting very high surface acidity. It is worth noticing that such removal of sulfate groups occurs at temperature values very close to those chosen for the calcination of the sulfated powder, i.e., 550 °C. This confirms that the detected sulfate content from TGA refers to strongly bounded groups on the oxide surface. 

Ion exchange capacity tests have been done on the powder to evaluate the number of acidic protons. The IEC estimated was 2.9 ± 0.09 meq/g, a very high value, even higher than that of Nafion itself, that is 0.9 meq/g (calculated as the reciprocal of the equivalent weight of the Nafion polymer here adopted). This confirms an outstanding acidity, due to the high sulfate groups content observed in the TGA analysis.

The value of the specific surface area of TiO_2_-SO_4_ powder, obtained by BET analysis, is 26 ± 1 m^2^g^−1^ and the sample shows the highest distribution of the pore size around 80 Å (data not shown here), revealing a dense mesoporous powder with a low concentration of free volume. The value of the surface area here detected is quite low with respect to other similar systems [21] and can be justified considering the high sulfur content, bound on the surface of the oxide, occupying the free volume of pores and creating strong polar interactions at the particles surface level.

### 3.2. Characterization of the Composite Membranes

The composition of the membranes here investigated and their thickness are reported in Table 1. The appearance of a typical composite membrane is also shown in the table. All the composite membranes results are opaque white, due to the inorganic filler particles, whereas plain Nafion (N) is transparent. All the membranes were realized with uniform thickness, measured in the dry state in the order of 100 µm.

**Figure 3:**
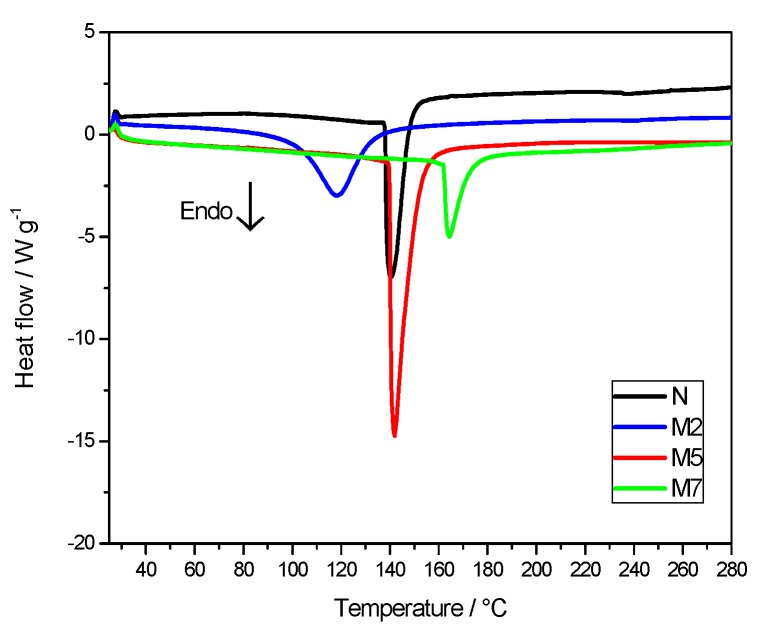
Differential scanning calorimetry (DSC) curves of the membranes.

The thermal response of Nafion membranes, with and without the inorganic additive, is displayed in Figure 3 in terms of DSC curves. In the temperature range under investigation, from 25 °C to 280 °C, one main endothermic peak is evident, associated to an order-disorder transition of the Nafion ionic clusters [25].

The enthalpy values, derived by the DSC peaks analysis, are reported in Table 2. Those of composite membranes M2 and M5 are higher with respect to M7 and N samples. As explained in the literature [26,27], this thermal transition, due to the polymer ionic clusters, is related to the hydration degree of the Nafion matrix. In particular, ΔH value increases, by increasing the water content, thanks to highly organized clusters and more cohesive interactions. At the same time, the change in the transition temperature can be attributed to a water-plasticizing effect, where lower transition temperatures may correspond to higher hydration levels [28]. In our case, the change in the enthalpy values is much more important than that in T_onset_. Clearly, the addition of hygroscopic sulfated titania particles causes an increase of the water content, resulting in very high ΔH values for M2 and for M5 with respect to plain Nafion. M7 sample, having the highest concentration of sulfated titania, apparently displays the lowest water affinity, likely due to phase segregation and non optimized distribution of the inorganic additive. Between all the composite membranes, M2 sample shows the lowest onset temperature associated to the thermal transition. 

**Figure 4:**
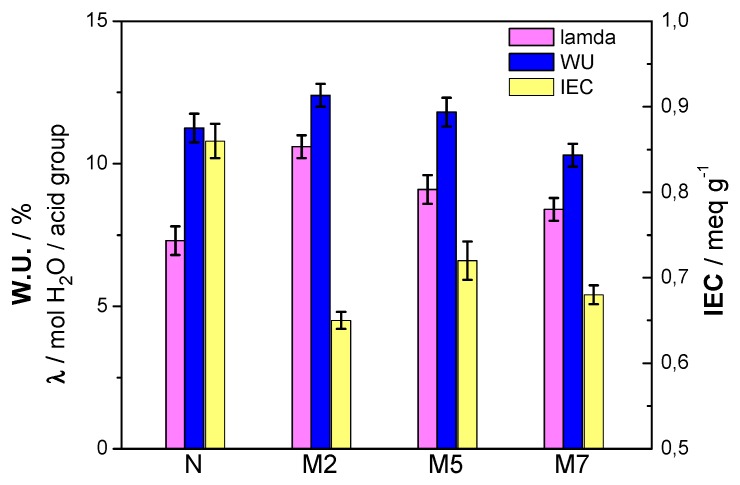
WU, IEC and λ of the membranes at 100 % RH.

Composite membranes were evaluated also in terms of water uptake (WU) and ion exchange capacity (IEC). These are important parameters because they provide a direct measure of the hydration level and of the number of available protons. Based on these parameters it possible to derived the value of λ [29]; the results are reported in Figure 4. The figure shows the composite membranes M2 and M5 exhibiting higher water content with respect to both plain Nafion and M7 sample, as also suggested by the DSC results. 

The Nafion polymer, used for this project, has an equivalent weight of 1100 geq^−1^ and, thus, its theoretical IEC value is 9 × 10^−4^ eqg^−1^. The ion exchange capacity obtained experimentally for the Nafion sample approaches this theoretical value. When the inorganic particles are added to Nafion, there is a significant decrease of the IEC. This could be related to an increase of density in the composite membranes and to the interaction of the additive with the polymer, decreasing the number of acid sites available per unit of mass. This leads to having a lower availability of exchangeable protons. However, the M5 sample has the highest IEC value among all the composite membranes. This critical effect of the filler content was already observed [29], with the intermediate additive concentration (5 wt %) being the optimal choice. Such effect can be explained considering a uniform distribution of the filler amount within the polymer matrix, establishing positive interactions in terms of proton exchangeability. With respect to lamda, all the composite membranes show higher values compared to plain Nafion. As expected, due to the high hydration, the M2 sample displays the largest concentration of water molecules per acidic group.

**Figure 5:**
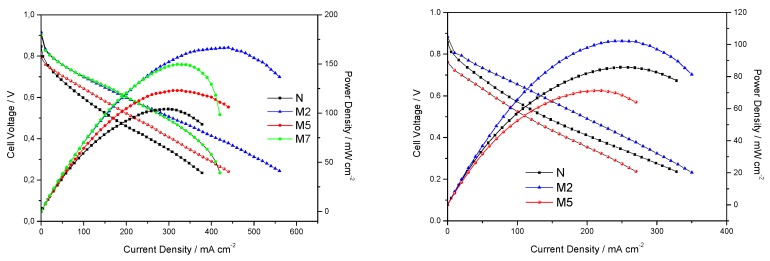
Fuel cell performances under 31% RH and at T = 80 °C (**on the left**) or at T= 110 °C (**on the right**).

Fuel cell performances were evaluated for all the membranes and reported in Figure 5. With the aim of testing the membranes under practical desirable conditions (low RH and high T), we recorded polarization and power-current curves under 31% relative humidity, moving from 80 °C to 110 °C. Better cell performances, in terms of maximum current and power delivered, are obtained when using the three composite membranes with respect to bare Nafion at 80 °C and 31% RH. Apparently, the most important contribution of the inorganic additive is seen in terms of reduced ohmic losses, with the composite membranes showing comparable slopes at the intermediate voltage-current range, lower than that associated to bare Nafion. The M7 sample, having the highest filler concentration, results in larger mass diffusion over-potentials, as evident from the slope changes at the highest current values (see Figure 5, left-side, green symbols). This again, as already discussed for DSC analysis, suggests a non-optimized morphology and filler distribution in the polymer-inorganic composite, hindering water/proton migration in M7 membrane. For this reason, M7 was not further adopted for fuel cell tests at higher temperature. So, the cell performances at 110 °C were evaluated only for N, M2 and M5 sample. When moving to 110 °C, as expected, the overall performance decreases, although M2 sample preserves the best result. Under these conditions, very important losses in the activation region are observed for M5, showing a low open circuit voltage. With respect to plain Nafion, quite a strange change in the slope of the polarization curve is detected, likely due to a difficult water management at such high temperature. To better elucidate the different contributions to the overall resistivity, in-operando impedance spectra were recorded while polarizing the cell at 0.6 V.

**Figure 6:**
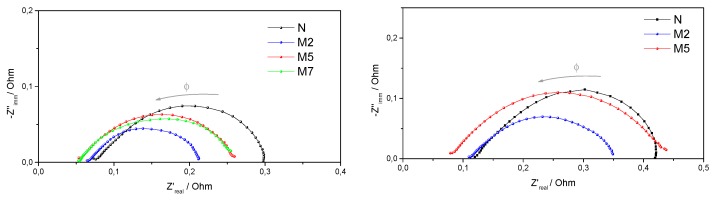
Impedance spectra recorded at 0.6 V, under 31% RH and at T = 80 °C (**on the left**) or at T= 110 °C (**on the right**). Frequency range, Φ: 10 KHz–1 Hz.

Nyquist plots of the impedance spectra, recorded during fuel cell operations, are reported in Figure 6. The high frequency intercept on the real axis is considered as the total non-electrode, ohmic resistance of the cell, meanwhile the low frequency intercept is mainly due to the electrode, charge-transfer resistance [30].

As expected from the fuel cell performances in Figure 5, the composite membranes give rise to lower ohmic resistances, confirming an improved proton conductivity due to the superacidic compound. Also, lower charge transfer resistances are observed at 80 °C, if compared to plain Nafion. At 110 °C, the M2 sample shows well controlled charge transfer resistance, allowing better cell performance. On the contrary, huge charge transfer resistances, much larger than Nafion itself, are associated to the M5 sample at high temperature, even though good proton conductivity is preserved, as evident from the intercept at high frequency. This explain the poor behavior of the M5 sample in the fuel cell test at 110 °C, where strong electrode polarizations were already observed. 

Overall, the inorganic additive is found to affect different important features of the composite membrane, that are (i) the proton conductivity, (ii) the hydration degree and (iii) the resistivity at the membrane/electrode interface. With respect to this latter point, a beneficial effect of the inorganic additive on the relaxation process of Nafion with temperature, avoiding or at least reducing shrinkage of the membrane, so ensuring a better interface contact with the electrodes, was already observed [31]. In this work, the contribution of sulfated titania in terms of cell charge transfer properties and electrode polarizations is found to play a crucial role at high temperature and is strongly dependent on the filler concentration. 

## 4. Conclusions

The properties of composite Nafion membranes with a sulfated titania additive were investigated. A new template-driven synthesis was developed to obtain highly acidic inorganic particles with mesoporous structure. They were prepared by a sol-gel process with a polymeric template agent, followed by hydrothermal heat treatment. The resulting powder has a low surface development with a very large sulfate content. From the TG analysis, the sulfate groups bonded on the oxide surface were found to be equal to 17% in weight. This value suggests high acidity of the powder and justifies its low surface area. In order to understand the role of the inorganic oxide in a polymer matrix, three composite membranes based on Nafion were prepared, with TiO_2_-SO_4_ nominal content of 2%, 5% or 7% w/w. 

The physical-chemical characterizations, performed on the composite membranes in comparison to plain Nafion, have shown how the inorganic additive has an enormous influence on both the ion-exchange capacity and the hydration ability of the membrane. Fuel cell tests, combined with impedance studies, at 31% RH and temperatures from 80 °C to 110 °C, revealed the beneficial effect of sulfated titania in terms of lower ohmic resistance and improved charge-transfer behavior, due to a better membrane-electrode interface. Membrane properties, as well as fuel cell performances, were found to be strongly dependent on the concentration of the filler, whose distribution within the polymer matrix is apparently critical. Among all the samples here investigated, indeed, the M2 membrane, with the lowest filler concentration, shows the best performance. 

This research demonstrated that the incorporation of a minimum amount of mesoporous sulfated titania can be a strategy to improve the low relative humidity, high temperature PEMFC performances.

## Figures and Tables

**Table 1:** Composition and thickness of the investigated membranes.

**Membrane**	**Nafion (wt.%)**	**TiO_2_-SO_4_ (wt.%)**	**Thickness (µm)**	**Picture of a typical composite membrane**
**N**	100		103 ± 5	
**M2**	98	2	107 ± 5	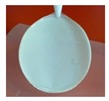
**M5**	95	5	98 ± 5	
**M7**	93	7	101 ± 5	

**Table 2:** ΔH and the T_onset_ associated to the DSC thermal transition of the polymer ionic clusters in the membranes (see Figure 4).

Membrane	ΔH [J g^−1^_polymer_]	T_onset_ [°C]
N	179.29	140
M2	223.74	118
M5	339.09	142
M7	111.18	164
